# Sorghum in dryland: morphological, physiological, and molecular responses of sorghum under drought stress

**DOI:** 10.1007/s00425-021-03799-7

**Published:** 2021-12-11

**Authors:** Kibrom B. Abreha, Muluken Enyew, Anders S. Carlsson, Ramesh R. Vetukuri, Tileye Feyissa, Tiny Motlhaodi, Dickson Ng’uni, Mulatu Geleta

**Affiliations:** 1 Department of Plant Breeding, Swedish University of Agricultural Sciences, Box 190, 234 22 Lomma, Sweden; 2 Institute of Biotechnology, Addis Ababa University, Box 1176, Addis Ababa, Ethiopia; 3 Department of Agricultural Research, Private Bag, 0033, Gaborone, Botswana; 4 Zambia Agriculture Research Institute, Mount Makulu Research Station, P/B 7, Chilanga, Zambia

**Keywords:** Drought tolerance, Germplasm, Grain quality, Sorghum, Source-sink relations

## Abstract

**Main conclusion:**

Droughts negatively affect sorghum’s productivity and nutritional quality. Across its diversity centers, however, there exist resilient genotypes that function differently under drought stress at various levels, including molecular and physiological.

**Abstract:**

Sorghum is an economically important and a staple food crop for over half a billion people in developing countries, mostly in arid and semi-arid regions where drought stress is a major limiting factor. Although sorghum is generally considered tolerant, drought stress still significantly hampers its productivity and nutritional quality across its major cultivation areas. Hence, understanding both the effects of the stress and plant response is indispensable for improving drought tolerance of the crop. This review aimed at enhancing our understanding and provide more insights on drought tolerance in sorghum as a contribution to the development of climate resilient sorghum cultivars. We summarized findings on the effects of drought on the growth and development of sorghum including osmotic potential that impedes germination process and embryonic structures, photosynthetic rates, and imbalance in source-sink relations that in turn affect seed filling often manifested in the form of substantial reduction in grain yield and quality. Mechanisms of sorghum response to drought-stress involving morphological, physiological, and molecular alterations are presented. We highlighted the current understanding about the genetic basis of drought tolerance in sorghum, which is important for maximizing utilization of its germplasm for development of improved cultivars. Furthermore, we discussed interactions of drought with other abiotic stresses and biotic factors, which may increase the vulnerability of the crop or enhance its tolerance to drought stress. Based on the research reviewed in this article, it appears possible to develop locally adapted cultivars of sorghum that are drought tolerant and nutrient rich using modern plant breeding techniques.

## Introduction

Myriads of biotic and abiotic stresses persistently challenge crops growing in the field under various environmental conditions. Because of global climate change, temperature and atmospheric CO_2_ levels are rising, and droughts are occurring more frequent and widespread. Drought is one of the most prominent abiotic stresses limiting crop production and productivity. It occurs recurrently in large parts of the world and affects all major crops. Severe drought substantially reduces crop yield and quality, and it can lead to famine in food insecure areas. However, crops differ in their tolerance to drought stress, and variation exists within a crop species.

Sorghum is a major staple crop for over half a billion people, mostly in developing countries in the semi-arid and arid tropics. It provides protein, fiber rich, and gluten-free nutrition (McCann et al. 2015; Impa et al. 2019). In addition to human nutrition, it is being used as a source of feedstock for bioethanol production (Mathur et al. 2017). Although sorghum is considered a drought-tolerant crop and can be productive under low-input conditions, drought stress due to water deficiency affects its soil-nutrient uptake capability and nutrient mobilization and transport (Yu et al. 2015; Sarshad et al. 2021). Sorghum is predominantly grown in semi-arid and arid areas, which are prone to water scarcity. For instance, 60% of the land in Sub-Saharan Africa where sorghum is commonly grown is considered vulnerable to recurrent droughts (Hadebe et al. 2017) and 80% of sorghum cultivated in the US is grown under non-irrigated conditions, where water is a major limiting factor, which substantially reduces yield (Crasta et al. 1999). Drought stress is regarded as the most frequent abiotic stress that sorghum faces in its major production areas (Assefa et al. 2010). As a result, considerable attention has been given to understand the effects of drought stress in sorghum and its stress tolerance mechanisms, as part of efforts to develop tolerant cultivars and apply efficient mitigation strategies in sorghum production.

Several studies have reported the impact of drought stress on sorghum. The stress affects sorghum growth and development from germination to reproductive and grain filling stages, as well as the plants’ physicochemical properties, which lead to substantial reduction in grain yield and quality (Kapanigowda et al. 2013; Sehgal et al. 2018; Bobade et al. 2019; Queiroz et al. 2019). Plant response to the stress involves changes in water use efficiency, transpiration rate, and remobilization of photosynthetic assimilates, as well as biochemical changes involving proline and other metabolites (Husen et al. 2014; Fracasso et al. 2016; Badigannavar et al. 2018; Zhang et al. 2019a). Stress response, which is associated with energy and fitness costs, and direct effects of the stress can devastate the whole crop but it is often manifested in the forms of significant loss in grain yield and reduction in nutritional quality (Fischer et al. 2019). Hence, drought stress could cause malnutrition in food-insecure and drought-prone areas where sorghum is a major crop. The effects of drought on sorghum nutritional quality is particularly interesting because inherently sorghum grain-protein has poor digestibility (Duodu et al. 2003), and drought can further reduce its digestibility (Impa et al. 2019) leading to poor nutrient absorption from consumed sorghum grown under drought stress.

Most of the recent review papers on sorghum-drought focus on specific topic, for instance on effect of the stress or plant response, indicating the need for a general review aimed to provide overview of the current knowledge and indicate gaps, and suggest its application in breeding programs. Here, we reviewed the previous and current studies about effects of drought on plant growth and development, grain filling, yield and nutritional composition in sorghum. We covered the crop’s physiological mechanisms and genetic basis of drought tolerance, and—omics studies employed to dissect the molecular basis of response to the stress. We have included data from our previous studies to highlight the possibility of targeting combination of traits in sorghum breeding for improved drought tolerance. As sorghum is concurrently challenged by a multitude of factors under different agricultural conditions, the interactions of drought with other abiotic stresses as well as biotic factors were also discussed. Furthermore, we highlighted knowledge gaps and possible challenges in the identification of drought-tolerant genotypes among the diverse sorghum germplasm for use in sorghum breeding programs targeting drought tolerance and broad adaptation.

## Impact of drought stress on growth and development of sorghum

In dryland areas where drought stress is prevalent, seedling death is a common problem, and is particularly high under combined drought and heat stress conditions, during seedling emergence and establishment (Ndlovu et al. 2021). Stand losses due to drought may occur after full emergence and before seedling establishment in sorghum (Queiroz et al. 2019). The early stage of plant growth (germination, emergence and seedling establishment) is potentially the most vulnerable to drought stress. As such, the impact of drought-induced water deficit on early developmental stages of sorghum has received significant attention. However, significant differences exist among sorghum genotypes in their response to varying degrees of drought-related stresses (Table 1).

**Table 1:** Summary of previously published results on the effects of drought stress on different sorghum genotypes at different stages

Experiment	Genotype name	No. of genotypes	Stress levels and conditions	Results	References
Evaluate the seed germination and early seedling growth under drought stress	Hybrid Dow 1G244	2	PEG 6000 (0.0; − 0.2; − 0.4; and − 0.8 MPa)	*Decrease*: germinated number of seeds, germination rate index, root and shoot length, shoot and root dry matter, and seedling vigor index. *Increase*: mean germination time and root: shoot ratio	(Queiroz et al. 2019)
Study the germination and early growth under drought stress	Sorghum genotypes	10	PEG 6000 (0, 20%) for 7 days in Petri dishes and for 21 days in a glass house	*Decrease*: plant height, seedling dry weight and the length or roots protruding *Increase*: proline level	(Chaniago et al. 2017)
Study the effect of drought, light intensity and heat stress on photosynthesis under field conditions	PA 59, E 36-1, IS 22380, M 35-1 and CSV 5	5	7.5% of PEG 6000 for 24 h or 48 h	*Increase*: chloroplastic chaperon, photosynthetic oxygen evolution in the resistance variety *Decrease*: photosystem II (PSII), PEPcase activity, Rubisco, chlorophyll content, fluorescence	(Jagtap et al. 1998)
Assess the impact of water stress on germination, emergence and growth at seedling stage	Jigurti, Gambella 1107, Meko, 76 T1 #23 and P9403	5	- PEG (0, − 0.20 and − 0.85 MPa) - Water contents (100, 60, 40 and 20% of field capacity)	*Decrease*: the rate of germination, percentage of germination, emergence, early seedlings growth, coleoptile, mesocotyl, radicle, and seedling shoot and root lengths, and root area	(Bayu et al. 2005)
Study the effect of water stress at germination and seedling stages	Forage sorghum (*Sorghum bicolor* cv. Speed feed)	1	- PEG 6000 (− 0.1, − 0.2, − 0.3, − 0.4, − 0.5, − 0.6, − 0.7, − 0.8, − 0.9, − 1, − 1.1, − 1.2, MPa) -Water stress (3, 6, 9 and 12 day)	*Decrease*: the percent and rate of seed germination, length of shoot and plant biomass	(Jafar et al. 2004)
Study the effect of drought stress at seedling and post-anthesis stages on morpho-physiological traits	sorghum genotypes/lines G-160, JS-61, H-118, H-18, PARC-SS-1, PGRI-141, PGRI-191, PGRI-29,PGRI-35 and JS-2002	10	Water stress: at 50% of the field capacity	*Decrease*: root length, coleoptile length, root: shoot ratio, flag leaf area, leaf dry matter, excised leaf weight loss, residual transpiration cell membrane stability and grain yield	(Ali et al. 2009)
Assess the genetic potential of different accessions to drought tolerance at seedling stage	Sorghum accessions	20	Irrigated daily with 50 ml of tap water per pot and PEG 6000 (0.0 MPa and − 1.03 MPa	*Decrease*: Shoot length, root length, fresh shoot weight, dry root weight, fresh root weight and dry root weight	(Bibi et al. 2012)
Examine the water and nitrogen utilization at seedling stage under extended drought stress	Commercial hybrid variety of sweet sorghum (*Sorghum bicolor* L. Moench, cv. Yajin19)		10% of PEG 6000 (− 0.3 M Pa) for 15 days	*Increase*: photosynthetic nitrogen-use efficiency, leaf dry mass per unit area *Decrease*: leaf area and total water loss per plant, total leaf nitrogen, transpiration rate, leaf nitrogen per unit area and leaf nitrogen concentration of stressed plants The dry mass of the whole plant was unaffected	(Wei-Feng and Yu-Zheng 2014)
Identify drought-tolerant sorghum landraces	South African sorghum landrace accessions and P898012, ICSV112	16	Water was withheld from pots for 6, 7, 8, and 9 days	- Higher chlorophyll and carotenoid contents for LR6 and LR35 in comparison to P898012 during severe stress - The proline content increased when the relative water content reduced - Identified four previously uncharacterized sorghum genotypes exhibiting drought tolerance	(Devnarain et al. 2016)
Evaluate the potential of accessions for drought tolerance and identify its physiological markers	Sorghum accessions	8	*Control*: irrigated daily with 50 mL *stressed*: withholding irrigation following planting	- *Reduction*: leaf stomatal conductance, osmotic potential, water potential, turgor pressure, shoot length and root length - *Increase*: root length under water stress conditions in some accessions - The osmotic potential was the highest contributor among all parameters used for drought tolerance	(Bibi et al. 2010)
Study the effects of drought stress on sorghum seedlings	SS-405, SX-17, Jumbo, and Revolution	4	30% PEG 6000 (24, 72 h)	- Higher level of CAT activity, APOD activity, GPOD activity, SOD activity, H_2_O_2_ content, MDA content, proline content, and chlorophyll content under stress condition - CAT activity increased under drought stress, particularly in the Jumbo and Revolution cultivars under both treatments - Both SX-17 and SS-405 showed high CAT activity at PEG for 72 h and low at 24 h	(Jung et al. 2020)
Investigate the effect of drought stress on quantity and quality of morpho-physiological traits	Sorghum varieties (Sepideh, Kimia, and Payam)	4	Control, preventing irrigation at pollination, seed milky, and seed doughy stages	- The pollination stage is more sensitive to drought stress - Drought stress negatively influenced morphological and yield-related traits, - Drought stress had positive effect on some quality-related traits such as total soluble carbohydrate, crude protein, and proline contents	(Sarshad et al. 2021)
Evaluate sorghum varieties for their genetic potential to drought under field conditions	Safal, BD 731, BD 740 and Hybrid Sorgo	4	Water stress (100%, 70%, 40% field capacity)	Water stress decreased plant height, no. of grain per panicle, thousand grain weight and no. of filled grain per hill No significant effect on panicle length and no. of unfilled grain hill-1	(Khaton et al. 2016)
Evaluate the response of genotypes to PEG induced drought stress and transcriptome analysis at seedling- stage	BTx623, SC56, Tx-7000 and PI-482662	4	PEG 8000 (− 0.5 MPa)	Reducted root length	(Abdel-Ghany et al. 2020)
Assessed water stress tolerance at seedling stages	Rabi sorghum	13	PEG 6000 (− 0, − 0.066, − 0.10, − 0.13 and − 0.16 MPa)	- Reduced percentage of seed germination, root length, RLSTI, shoot length, SLSTI, seedling dry weight, SDWSTI and seed vigour - Genotype CSV-29R, Elongvan-19, M 35-1 and Phule Maulee showed comparatively better performance in drought stress	(Bobade et al. 2019)

Drought tolerance genotypes = BTx623, SC56, PA 59 from Cameroon, E 36-1 from Ethiopia, IS 22380 from Sudan, M 35-1 from India, P898012, Payam, Kimia. Drought sensitive genotypes = Tx-7000, PI-482662, CSV 5 from India, ICSV112 from the International Crops Research Institute for the Semi-Arid Tropics (ICRISAT), India

Semi-sensitive to stress = Sepideh. SS405 = sorghum forage hybrid; SX-17 and Jumbo = sorghum-sudangrass hybrids; Revolution = hybrid of brown midrib. Safal, BD 731, BD 740 (indigenous) were collected from Bangladesh Agricultural Development Corporation and Bangladesh Agricultural Research Institute whereas Hybrid Sorgo, an exogenous variety, was collected from Japan. Rubisco = ribulose-1,5-bisphosphate carboxylase; PEPcase = phosphoenolpyruvate photosynthesis; MDA = malondialdehyde; GPOD = Guaiacol peroxidase; SOD = Super oxide dismutase; H_2_O_2_ = hydrogen peroxide

Different studies have shown that induced drought stress through polyethylene glycol (PEG) significantly reduces rate and percentage of seed germination in sorghum (Jafar et al. 2004; Bayu et al. 2005; Bobade et al. 2019; Queiroz et al. 2019). Similarly, water deficit at different levels of soil water content (60% and 40% field capacity) significantly reduced percentage of seed emergence (Bayu et al. 2005). Decreasing the osmotic potential level from zero to − 0.8 MPa significantly reduced percent germination (PG), and germination rate index (GRI) as well as the amount of water absorbed by seeds (Oliveira and Gomes-Filho 2009). The GRI was significantly greater in high osmotic potential environment, and the mean germination time (MGT) was longer under lower osmotic potential environment. Drought stress affects starch synthesis and energy ((adenosine triphosphate (ATP)) production process through increased respiration rate, resulting in reduced index of seedling vigor, GRI and PG (Queiroz et al. 2019). Research showed that differences in starch content exist among sorghum genotypes. However, differences among these genotypes’ adaptive mechanisms and how the water deficit affects starch biosynthesis during seed germination has not been thoroughly investigated. The delay in germination was shown to be the result of a highly negative osmotic potential that negatively affected the water uptake of the seeds, i.e., imbibition, which is the first step of the germination process (Queiroz et al. 2019). For a successful germination, seeds should reach an adequate level of hydration during the imbibition phase, to reactivate the seed metabolic processes and stimulate the growth of embryonic axis. Plants subjected to severe drought stress require more time to adjust the internal osmotic potential in accordance with the external environment.

After germination, drought stress can significantly reduce radicle, hypocotyl, and plumule (including coleoptile and mesocotyl) growth (Bayu et al. 2005; Reiahi and Farahbakhsh 2013; Queiroz et al. 2019). According to Queiroz et al. (2019), inhibition of radicle emergence and growth under water deficit condition could be due to impairment of cell division and elongation resulting from a reduction in the turgor of the radicle cells. This could affect the subsequent stages of plant growth and development. For example, Bayu et al. (2005) reported that the length of the coleoptile and mesocotyl was reduced under mild and severe water deficit conditions. The mesocotyl and coleoptile are essential for successful emergence and early plant vigor. Poor elongation of mesocotyl and coleoptiles under water-deficit conditions implies poor seedling emergence and establishment. Furthermore, water deficit significantly reduces shoot elongation and dry weight, and to some extent root growth (Takele 2000; Jafar et al. 2004; Bobade et al. 2019; Queiroz et al. 2019). Similarly, Bayu et al. (2005) showed an increase in the root to shoot ratio and the rise of osmotic potential levels, probably as an adaptive response to water-deficit conditions. Furthermore, the decrease in shoot growth rate could be due to a reduction in one or both of the primary cellular growth parameters: wall extensibility and cell turgor (Queiroz et al. 2019).

Studies have shown that the effect of water deficit on vegetative growth is more pronounced on drought-sensitive than drought-tolerant sorghum cultivars. In a study by Fadoul et al. (2018), under drought stress conditions shoot and root length were reduced in the drought-sensitive cultivar compared to the drought-tolerant genotypes. This suggests that cultivars that can establish long and extensive root systems may have more successful seedling establishment as their root systems can rapidly penetrate the upper soil layers and reach moist soil layers for water uptake, thus mitigating the stress due to water deficit. Hence, it is crucial to consider traits at the early stage of plant growth and development, such as seed vigor, imbibition, germination potential, germination rate, plumule and radicle development as well as root and shoot growth when screening for drought-tolerant sorghum genotypes.

## The effect of drought stress on sorghum grain yield

Drought stress, explained by soil water deficit, is one of the major abiotic stresses severely affecting crop yield worldwide. Drought stress has a capacity to significantly delay floral initiation, and affect panicle development and appearance of new leaves (Ndlovu et al. 2021). It reduces photosynthesis, chlorophyll content (Soil Plant Analysis Development; SPAD), translocation of photo assimilates, and soil nutrient uptake resulting in reduced grain yield and quality (Assefa et al. 2010; Kapanigowda et al. 2013; Sehgal et al. 2018). Crops respond to drought stress conditions to varying extents through different mechanisms. Drought tolerance involves physiological and molecular mechanisms (Sabadin et al. 2012) for activation of relevant genes and pathways, allocation of energy and resource for cellular functioning, and modification in stomatal conductance and transpiration as well as for increasing water use efficiency and promoting stay-green (Tovignan et al. 2020). Thus, drought tolerance is a result of diverse physico-chemical alterations (Sehgal et al. 2018; Impa et al. 2019) leading to fitness costs that hampers crop productivity. Drought stress substantially affect grain yields by reducing seed size, number and grain weight per panicle as well as other agronomic traits (Sehgal et al. 2018; Sarshad et al. 2021). In short, it increases both direct and indirect costs to crops, which limit their production and productivity.

Studies have shown that sorghum is one of the best drought-tolerant crop adapted to diverse agro-ecology and low-input agriculture, but still drought stress can cause significant yield losses (Assefa et al. 2010; Sabadin et al. 2012) even in drought-tolerant cultivars (Ray et al. 2018). This can be regarded as the fitness cost of the tolerance mechanisms manifested itself as a loss in grain yield. In water insecure areas, erratic and insufficient precipitation often substantially reduces grain yield (Hattori et al. 2005). This holds true even when the drought stress occurs at the seedling stage (Gano et al. 2021) suggesting that drought stress can reduce grain yields at any stage of crop development. However, almost all previous studies have focused on the effect of the stress occurring during a specific developmental stage although, under natural conditions, the stress is consistently present across several stages. Drought stress at the vegetative and reproductive stages reduced grain yield by more than 36% and 55%, respectively (Assefa et al. 2010). The stress imposed during booting and flowering stages caused 87% reduction in grain yield, but only significantly longer and more intense drought stress at vegetative stage can lead to such a substantial yield loss (Crafurd and Peacock 1993). Hence, although drought stress at any developmental stage can affect grain yield, the stress during reproductive stages has a more drastic effect on grain yield. This is because there is a stronger relationship between the environment and grain yield and quality during reproductive stages than at the earlier vegetative stages. Reproductive stages such as flowering, pollination, microsporogenesis, and seed filling (Sarshad et al. 2021) were shown to be critical that can adversely affect grain yield (Kebede et al. 2001). Particularly, seed filling, which involves a number of metabolic processes, diverse enzymes, and transporters located in the leaves and seeds, is considered the most sensitive stage to drought stress (De Souza et al. 2015; Sehgal et al. 2018).

The production of sorghum is affected by drought stress during both pre-flowering (panicle development) and post-flowering stage (between flowering and grain development) (Adugna and Tirfessa 2014). A study on sorghum by (Kapanigowda et al. 2013) showed that both pre- and post-flowering drought stress significantly reduces grain quantity and quality. The occurrence of drought stress during flowering stage can also cause a reduction in number of grains per panicle (Manjarrez-Sandoval et al. 1989), which is a trait directly contributing to grain yield. However, a drought during post-flowering stages has a more severe impact on grain yield compared to a drought during pre-flowering stages. For example, sorghum growers in Ethiopia and Burkina Faso indicated that severe drought during post-flowering stages is a major sorghum production constraint (Ouedraogo et al. 2017; Derese et al. 2018). Similarly, Burke et al. (2018) reported that drought stress during the post-flowering growth stage had drastic effects on sorghum productivity due to premature plant death and reduced seed size (Burke et al. 2018). A classical study over a period of 2 years involving 30 sorghum cultivars showed that drought stress during post-flowering stage reduced grain yield by about 50% (Batista et al. 2019). However, the effect of drought stress on different sorghum genotypes may differ due to the variability in their response to the stress. For example, drought stress during terminal post-flowering stage, genotypes with a high growth rate and short duration of grain filling produced larger grains compared to genotypes with longer duration of grain development (Tuinstra et al. 1997). Metabolic and enzyme assays are required to understand how water deficit affect starch synthesis and subsequent germination and metabolic response of sorghum seeds. Drought stress at pollination stage can lead to significant decrease in grain yield because of the deficiency of insemination of eggs inside the ovary (Sarshad et al. 2021). This is related to the fact that the transfer of pollen grains from male to female organs and contact with the eggs in the ovary require sufficient moisture, which is a limiting factor under drought stress conditions. In line with this, Manjarrez-Sandoval et al. (1989) reported that severe drought stress prior to microsporogenesis caused a decrease in grain number per panicle (but with slight increase in grain size), which subsequently led to lower grain yield. On the other hand, a study by Sarshad et al. (2021) showed that drought stress after grain filling have no significant adverse effect on gain yield. Although not experimentally validated, the effect of the stress after grain filling might affect moisture content and alter the metabolism in the mature seeds resulting in transgenerational effect, particularly germination rate and early seedling of offspring. Overall, research clearly showed that drought stress reduces grain yield; however, the impact level of the stress depends on several factors. Stress intensity and duration, development stage and genotype of the plants, presence of other confounding stresses and seasonal variations contribute to the differences in the magnitude of the damage caused by the drought stress. This is particularly important because drought tolerance is a multigenic trait, and hence these environmental conditions may led to pronounced variability among the results reported.

## The effect of drought stress on nutritional quality

Drought stress alters the relationship between morpho-physiological traits on hand and the source activity and sink strength on the other (Yu et al. 2015), alters grain physico-chemical characteristics (Impa et al. 2019), and reduces mineralization of nutrients and impair membrane permeability (Stagnari et al. 2016). In line with these changes in plants due to drought stress, various studies have shown its effect on nutritional content and composition in sorghum. For example, growing sorghum genotypes under dry conditions led to decreased grain micronutrient content (Zn, Fe, Mn and Cu) (Impa et al. 2019). Similarly, drought stress induced during flowering stage led to reduced total starch, amylase and amylopectin accumulation, which is related to compromised activities on sugar nucleotide precursors by enzymes, such as starch synthase (SSS), granule-bound starch synthase (GBSS), starch branching enzyme (SBE), and starch debranching enzymes (DBE) during grain filling (Yi et al. 2014). On the other hand, Ananda et al. (2011) reported that drought stress imposed on sorghum at different phenological stages, from flowering to late seed filling, did not significantly affect glucose content and concomitant ethanol production. However, the plants were subjected to the stress at each specific phenological stage separately, and hence the results could not be conclusive since this scenario might not exist under natural field conditions. The response of plants to water deficit involves activation of myriad of signaling pathways with a phytohormone abscisic acid (ABA) playing a central role and leading to biosynthesis accumulation of some metabolites (amino acids, sugars, indoles, phenolics, and glucosinolates) mainly in drought-tolerant genotypes (Stagnari et al. 2016). For example, there was higher grain K and Fe concentrations in drought-tolerant genotypes compared to the susceptible ones (Abu Assar et al. 2002). In a study by Ogbaga et al. (2016), drought-induced stress led to increased sugar and sugar alcohol contents in a sorghum genotype (Samsorg 17) and amino acid concentration in another sorghum genotype (Samsorg 40). Other studies on sorghum showed that drought stress increased the total protein content and positively affected the total soluble carbohydrate, crude protein, and proline contents (Impa et al. 2019; Sarshad et al. 2021). The increased amount of these molecules indicates their role in drought stress tolerance, whereas the differences in the level of their accumulation among genotypes points to genotype-specific tolerance mechanisms. Drought stress could also influence nutrient availability. For example, Impa et al. (2019) reported decreased digestibility of protein obtained from sorghum grown under drought stressed conditions. This could be associated with increased level of starches, as a response to the drought stress (Stagnari et al. 2016).

**Fig. 1:**
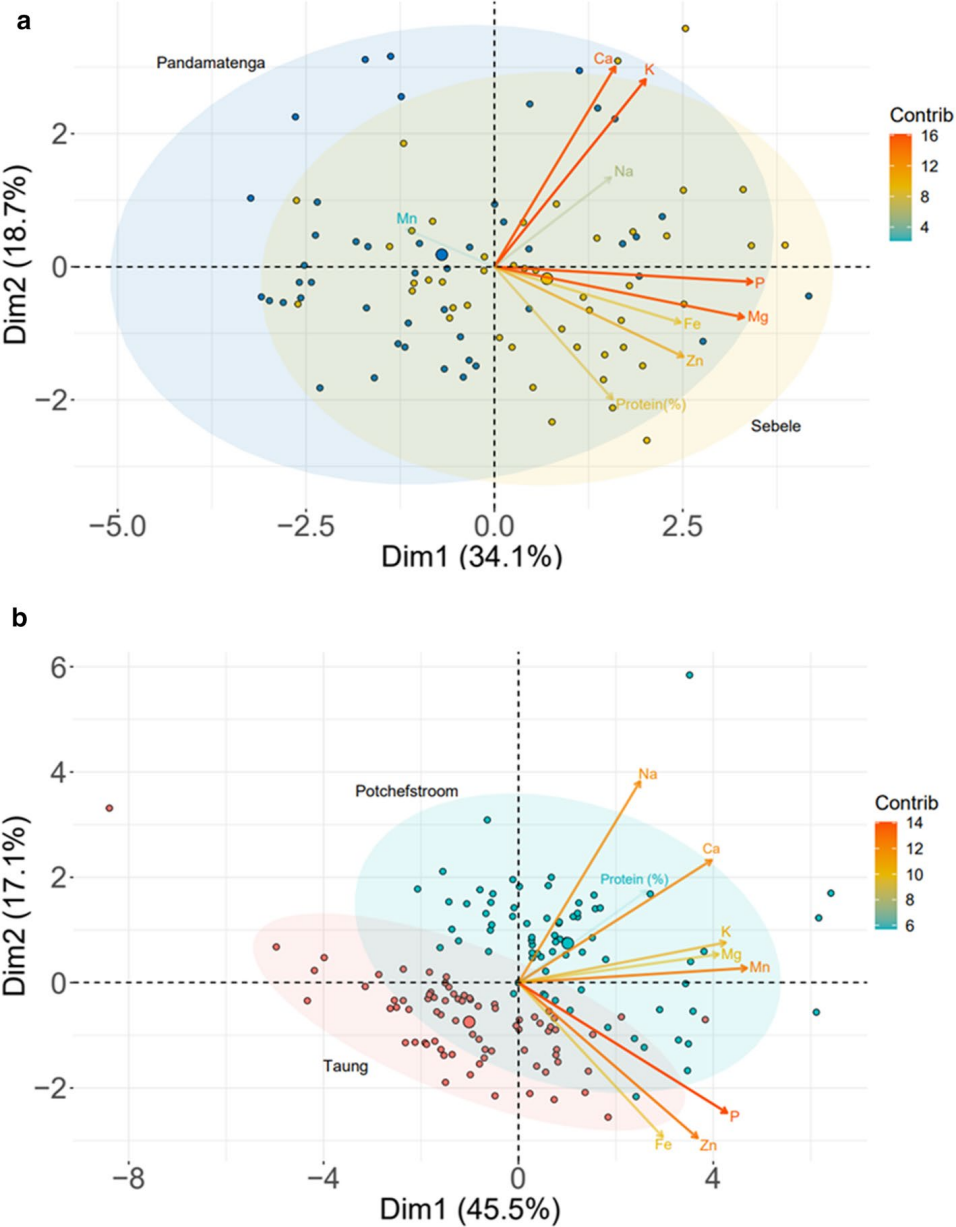
PCA-biplot of sorghum genotypes grown **a** at two locations in Botswana in 2015 (Motlhaodi et al. 2018) and **b** at two locations in South Africa in 2011 (Ng’uni et al. 2016). The two studies used different sorghum genotypes

A highly significant variation exists among sorghum genotypes in grain nutrient contents. Total starch, amylose and mineral content varied significantly among sorghum varieties where two varieties (Tx430 and AR-3048) were found to contain significantly higher protein content than others (Ng’uni et al. 2016). Motlhaodi et al. (2018) reported significant differences among sorghum accessions for protein and nutrients (Ca, Fe, K, Mg, Mn, Na, P and Zn) and these traits have a strong broad-sense heritability ranging from 0.62 to 0.85. Similarly, analysis of 336 sorghum recombinant inbred lines (RILs) showed large variability and high heritability for Fe and Zn content (Phuke et al. 2017). Nevertheless, these studies did not investigate the effect of drought stress on the concentrations of these nutrients in different sorghum genotypes. However, Abu Assar et al. (2002) reported that sorghum genotypes showed considerable variation in mineral composition, with drought-tolerant genotypes containing higher K and Fe content compared to susceptible ones when grown under drought stress conditions. Thus, the tolerant genotypes could maintain optimal mineral and other nutrient compositions even when grown under water deficit conditions. The presence of genotypes with higher concentration of Fe and Zn and stable heritability of the nutrient content (Motlhaodi et al. 2018) suggest sorghum genotypes with higher concentration of these nutrients can be utilized for enhancing micronutrient composition in elite sorghum materials. The strong positive correlation among micronutrients (Ng’uni et al. 2016; Phuke et al. 2017) and their high heritability in some genotypes indicate strong genetic control of these important quality traits. To evaluate performance of sorghum accessions and identify traits that are correlated across environments, we have obtained the raw data from previous studies in South Africa (Ng’uni et al. 2016) and Botswana (Motlhaodi et al. 2018), filtered the parameters that were measured in both studies and performed PCA analysis in R statistical software version.4.0.3. These studies were conducted on different sorghum genotypes grown in different years and locations but both studies showed strong correlation among K, Ca, and Na as well as among Fe, Zn, and P (Fig. 1). This indicates several quality traits can be targeted for a combined genetic improvement in sorghum. But the composition of these micronutrients in sorghum could vary at different maturity stages (Abu Assar et al. 2002). This could be due to changes in nutrient demands of the plants or nutrient availability in the soil, as well as other coexisting biotic and abiotic factors. Hence, gaining a deeper understanding of the inheritance of these nutrients and the key genes involved in nutrient accumulation, particularly in sorghum grown under drought stress conditions, is crucial for enhancing these grain quality traits and help alleviate malnutrition.

## Mechanisms of drought tolerance in sorghum

### Physiological mechanisms of drought tolerance in sorghum

**Fig. 2:**
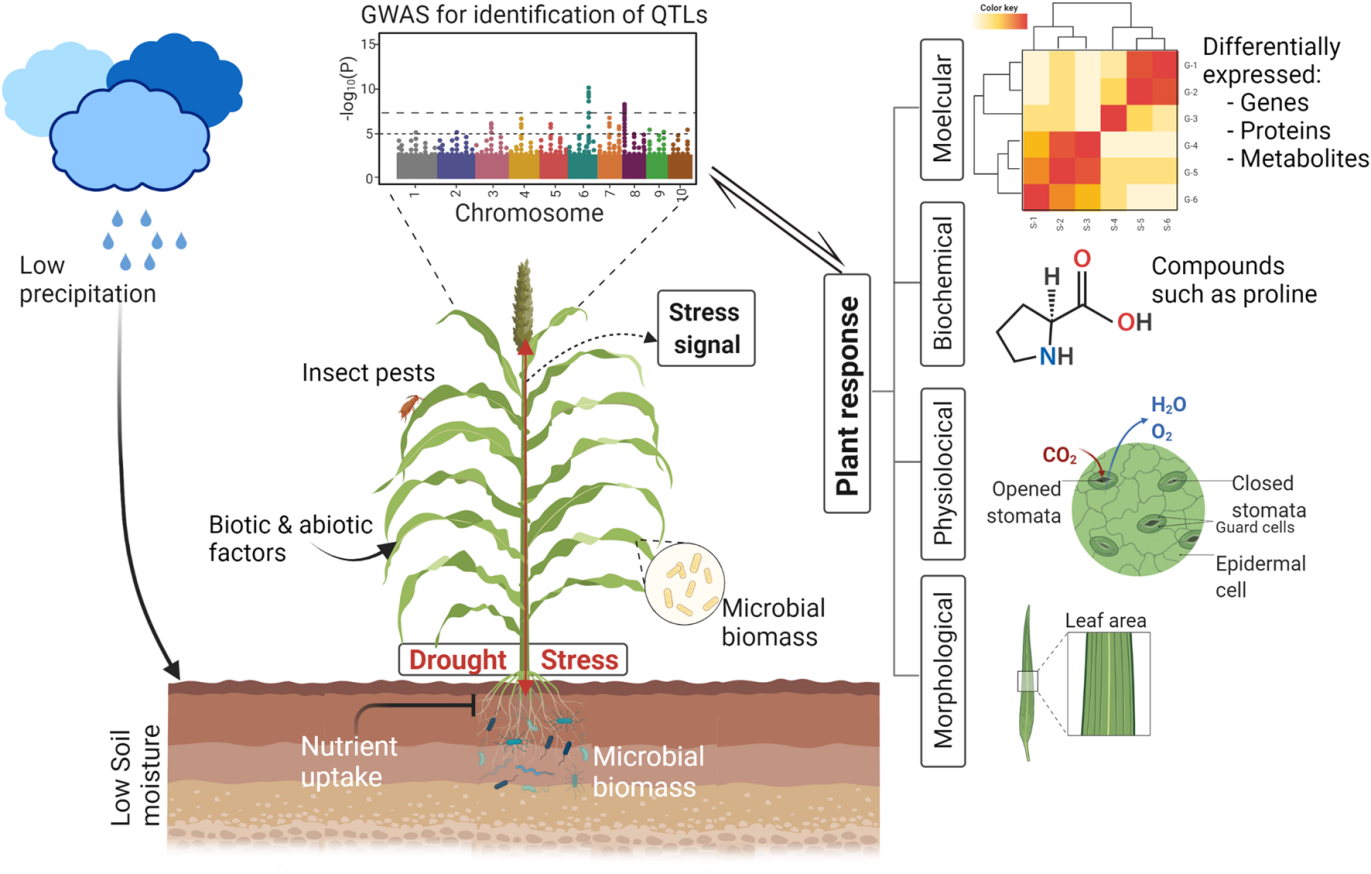
Diagrammatic depiction of morphological, physiological, biochemical, and molecular responses of sorghum to drought stress. This figure was created with BioRender (https://biorender.com/)

Plant response to drought stress and drought tolerance is a result of complex biological processes involving physiological, biochemical, genomic, proteomic and metabolomics changes (Ngara et al. 2021). An overview of drought stress and sorghum plant responses is provided in Fig. 2. Plants mitigate effects of drought through avoidance, recovery, survival, and tolerance mechanisms. Drought avoidance is the ability of plants to conserve water through reducing water loss from the shoots or by more effectively extracting water from the soil (Ludlow and Muchow 1990; Osmolovskaya et al. 2018). Moreover, plants survive drought stress by extending their root system, stomatal closure, leaf rolling, stem waxiness, stay green and high transpiration efficiency (Badigannavar et al. 2018). The more effective mechanism is drought escape, which refers to plants’ drought avoidance by completing their life cycles before the onset of a dry period to sustain reproduction (Manavalan and Nguyen 2017). The drought escape mechanisms are early flowering and maturity, high leaf N_2_ level, high photosynthetic capacity and remobilization of assimilates (Badigannavar et al. 2018). Whereas, drought tolerance is the ability of plants to withstand water stress while keeping vital physiological activities that stabilize and protect metabolic integrity at tissue and cellular levels (Tuinstra et al. 1997). This can be osmotic adjustments, protective solutes, high proline, desiccation tolerant enzymes and high stomatal conductance (Badigannavar et al. 2018).

### Photosynthetic rate, transpiration and stomatal conductance

In drought sensitive sorghum genotypes, stress-induced physiological modifications such as change in rate of photosynthesis were observed under stress conditions (Fracasso et al. 2016). Drought stress has significant effects on photosynthetic rate (A), transpiration rate (E), water use efficiency (WUE) and stomatal conductance. The Fv/Fm, which refers to maximum quantum yield of photosystem II is an important tool for measuring the impact of drought stress on photosynthesis (Husen 2010; Husen et al. 2014). It is used as an indicator of the level of photosynthetic efficiency, which is significantly lower in sorghum grown under drought stress conditions (Johnson et al. 2014). Drought stress affects photosynthetic rate in sorghum by decreasing stomatal conductance and transpiration rate (Zhang et al. 2019b), quantum yield and increasing leaf temperature (Kapanigowda et al. 2014), reduction in chlorophyll and Rubisco, increase in O_2_ evolution and decrease in PEPCase activity (Bao et al. 2017). Different studies have shown that, under drought stress conditions, tolerant sorghum genotypes have significantly higher values of Fv/Fm and photosynthetic rate (Fracasso et al. 2016; Sukumaran et al. 2016). In addition to plants’ ability to avoid and/or tolerate drought stress, photosynthetic recovery that comes following rehydration plays an important role in dictating their tolerance to drought as well as in preventing reduction in grain yield (Chaves et al. 2009). Increased photosynthetic rate that provides raw material and energy required for growth and development under drought stress is a major mechanism through which tolerant genotypes maintain grain yield in sorghum (Getnet et al. 2015).

Several studies have indicated significant genetic variation in sorghum in terms of net carbon assimilation rate (A), transpiration rate (E), A:E ratio and WUE under normal and drought stress conditions. Different studies reported that a rise in A:E and WUE improve drought tolerance during pre-flowering stage in sorghum (Balota et al. 2008; Vadez et al. 2011b). In drought-tolerant sorghum genotypes, transpiration efficiency did not differ between the control and drought stressed plants while there was a statistically significant difference between the control and drought stressed plants in the case of drought sensitive genotypes (Fracasso et al. 2016). Furthermore, drought-tolerant genotypes showed a significantly higher WUE than drought sensitive genotypes during drought stress period (Fracasso et al. 2016). Transpiration efficiency and water extraction were reported to be significantly associated with grain yield in sorghum (Vadez et al. 2011b). A study by showed that the average heritability estimates for A:E were 0.9 at 40% field capacity (FC) and 0.8 at 80% FC indicating strong genetic basis for the trait. Thus, given the role of genetic basis of the tolerance mechanisms (Fig. 2), selection of desirable genotypes based on this trait is important for developing drought-tolerant sorghum genotypes. Genotypes with reduced stomatal conductance and reduced transpiration rate (E) throughout the vegetative phase conserve water that may be used during grain filling stage in water-limited environments (Lopez et al. 2017), and hence can be categorized as drought tolerant. This interesting study by Lopez et al. (2017) showed that QTL identified for stomatal conductance was associated with reduced E but not A or shoot biomass.

### Chlorophyll content and stay green

The ability of a plant to maintain normal chlorophyll content under drought stress conditions contributes to its drought adaptability (Chen et al. 2016). The total chlorophyll content as well as chlorophyll a and b contents directly affect the plant capacity to absorb light for photosynthesis. Different studies have reported a significant reduction in chlorophyll content in sorghum grown under drought stress (Xu et al. 2000; Reddy et al. 2014; Fracasso et al. 2016; Fadoul et al. 2018; Amoah and Antwi-Berko 2020). For instance, there was 23% reduction in total chlorophyll content in a stay green genotypes and 75% reduction in senescent genotypes grown under drought stress conditions, compared to corresponding genotypes grown under control conditions (Xu et al. 2000). Another study reported 4.3% reduction in total chlorophyll content in stressed plants as compared to the control plants (Devnarain et al. 2016). In addition to chlorophyll, decreased concentration of some carotenoids under severe drought stress conditions has been reported (Munné‐Bosch et al. 2001). Takele (2010) reported that drought tolerance reduced both chlorophyll and carotenoid contents during pre- and post-flowering stages in drought-tolerant sorghum. A decrease in carotinoids is probably due to the down-regulation of genes involved in the terpenoid and carotenoid biosynthesis in drought sensitive genotypes (Fracasso et al. 2016). Drought stress induces down-regulation of genes related to carotenoids and chlorophyll biosynthetic pathways, which drastically affects light reaction and carbon fixation pathways. The chlorophyll content at maturity had a significant positive correlation with green leaf number and green leaf area at flowering and maturity stages. In turn, both leaf traits at both stages correlated significantly with grain yield (Reddy et al. 2014).

Leaf senescence is characterized by a loss of chlorophyll and progressive decline in photosynthetic capacity (Borrell et al. 2000; Tao et al. 2000). Stay-green is a well-characterized trait contributing to the adaptation of sorghum to post-flowering drought conditions that confers delayed leaf senescence and improved grain yield. Several scholars have tried to understand the physiological mechanism of stay green in sorghum. Early hypotheses and studies suggested that stay green is associated with a higher leaf nitrogen concentration, cytokinin and chlorophyll content under drought stress conditions. For example, Borrell et al. (2000) reported the association of stay green with higher leaf nitrogen concentration, mainly at flowering stage. Another study showed that stay green sorghum genotypes maintain high levels of cytokinin indicating a lower senescence rate of the stay green genotypes (Thomas and Howarth 2000). Furthermore, the stay green genotypes show higher levels of chlorophyll content than senescent genotypes (Xu et al. 2000).

The stay green trait of sorghum as a response to drought is associated with higher leaf chlorophyll content, slower rate of loss of green leaf area (Kassahun et al. 2010), decreased tillering and size of upper leaves (Borrell et al. 2014a, b; George-Jaeggli et al. 2017). The trait is also linked with increased transpiration efficiency (TE) and water extraction (Vadez et al. 2011a). Under drought stress conditions, the introgression of stay green QTLs from B35 to senescent variety R 16 showed higher leaf chlorophyll levels at flowering and a greater percentage green leaf area during grain filling stages and associated with a higher relative grain yield during the post-flowering stages (Kassahun et al. 2010). The *Stg* QTL control canopy size in the form of reduced tillering and the size of upper leaves, enlarged size of lower leaves, and in some cases reduced number of leaves per culm, at flowering stages. The reduced canopy size at flowering decreases pre-flowering water demand, thereby leads to increased water availability during grain filling stage, which in turn lead to increased biomass production and grain yield (Borrell et al. 2014a, b). Accelerated age-related senescence of lower leaves in stay green lines before flowering results in shedding of old leaves at flowering stage thereby contributes to having smaller canopy (George-Jaeggli et al. 2017). Any water savings during pre-flowering stages increases water availability during post-flowering stages, which allows plants retain photosynthetic capacity for longer period of time and “staying green” during grain filling (George-Jaeggli et al. 2017). Based on these and related research results, stay green could be considered as a post flowering drought tolerance mechanism that facilitates the availability of water required for overall growth and grain production.

## The genetic basis of drought tolerance traits

### Association mapping for drought tolerance traits

Many important traits for drought tolerance like stay green, chlorophyll content, leaf number, leaf length, leaf width and leaf area as well as root traits are controlled by multiple genes located within genomic regions referred to as quantitative trait loci (QTLs). The identification and understanding of the QTLs associated with these traits are highly important for the development of drought-tolerant sorghum cultivars. A number of drought related traits have been identified and several QTLs associated with these traits were mapped in sorghum (Table 2). However, most of these QTLs were identified using bi-parental linkage mapping. In this regard, future studies should focus on genome wide association mapping with high dense SNP markers to accurately identify QTLs associated with the traits. Among drought tolerance related traits in sorghum, stay-green, which is associated with chlorophyll content is the best characterized, and is considered a very important trait for sustainable grain yield under drought stress particularly during grain filling period.

**Table 2:** Quantitative trait loci (QTLs) mapped for drought tolerance related traits in sorghum

Trait	No./Population	No of markers	Type of Markers	Chr/LG	Method^a^	Env^b^	References
Stay green	98 RI (TX7078 and B35)	170	RAPD, RFLP	B, F, G, H, I	LM	DS	(Tuinstra et al. 1997)
	248 RILs (Tx436 and 00MN7645)	7144	SNP	4, 5, 6, 7, 10	LM	DS	(Sukumaran et al. 2016)
	160 RILs (QL39 and QL41)	2	SSR, RFLP	A, B, C, G, I	LM	DF	(Tao et al. 2000)
	96 RILs (B35 and Tx430)	142	RLFP	A, B, D, J, I	LM	DS	(Crasta et al. 1999)
	98 RILs (B35 and Tx7000)	142	RLFP	A, D, J	LM	DS	(Xu et al. 2000)
	2000 NIL (BTx642 and RTx7000)	113	AFLP, SSR	A, D, J	LM	DS	(Harris et al. 2007)
	125 RILs (SC56 and Tx7000)	170	RFLP	G, J, C, B, D, F	LM	DS	(Kebede et al. 2001)
	98 RILs (B35 × Tx7000)	91	RFLP, SSR, RAPD	A, D, J	LM	DS	(Subudhi et al. 2000)
	226 RILs (IS9830 × E36-1 and N13 × E36-1)	225	AFLP, SSR, RFLP, RAPD	A, E, G	LM	DS	(Haussmann et al. 2002)
	100 RILs (BR007 and SC2839)	344	DArT, SSR, STS, RFLP	3, 4, 8, 10	LM	DS	(Sabadin et al. 2012)
Chlorphyll content at flowering	245 RILs (M35-1 and B35)	237	SSR and morphological	9, 1, 3, 5, 6, 7	LM	DS	(Reddy et al. 2014)
Chlorphyll content at maturity	245 RILs (M35-1 and B35)	237	SSR	1, 2, 7, 9, 10	LM	DS	(Reddy et al. 2014)
	98 RILs (B35 and Tx7000)	142	RLFP	A, D, J	LM	DS	(Xu et al. 2000)
	188 RILs (Tx436 × 00MN7645	7144	SNP	4	LM	DS	(Sukumaran et al. 2016)
	70 RILs (Tx430 and Tx7078)	261	SNPs	4	LM	DS	(Kapanigowda et al. 2014)
Green leaf number at flowering	245 RILs (M35-1 and B35)	237	SSR	1, 2, 3, 4, 9	LM	DS	(Reddy et al. 2014)
Green leaf number at maturity	245 RILs (M35-1 and B35)	237	SSR andmorphological	1, 2, 3, 4, 7, 9	LM	DS	(Reddy et al. 2014)
Total leaf number	168 RILs (296B and IS18551)	152	SSR, morphological	1, 3, 7	LM	DS	(Srinivas et al. 2009)
Leaf number	70 RILs (Tx430 and Tx7078)	6128	SNP	6	LM	DS	(Lopez et al. 2017)
	184 RILs (E36-1 × SPV70)	104	EST-SSR, SSR, SNP	1, 10	LM	DS	(Fakrudin et al. 2013)
Percent green leaves retained at maturity	245 RILs (M35-1 and B35)	237	SSR, morphological	1, 2, 3, 4, 7, 9	LM	DS	(Reddy et al. 2014)
	226 RIP (IS9830 × E36-1) and N13 × E36-1)	128	AFLP, RFLP, SSR, RAPD	A, D, G, H, B, C, E	LM	DS	(Haussmann et al. 2002)
Green leaf area at flowering	245 RILs (M35-1 and B35)	237		1, 2, 3, 9	LM	DS	(Reddy et al. 2014)
	168 RILs (296B and IS18551)	152	SSR, morphological	1, 3, 4, 5	LM	DS	(Srinivas et al. 2009)
chlorophyll fluorescence (F_v_/F_m_)	188 RILs (Tx436 × 00MN7645)	7144	SNP	3, 4	LM	DS	(Sukumaran et al. 2016)
	226 RIP (IS9830 × E36-1) and N13 × E36-1)	28	AFLP, RFLP, SSR, RAPD	C, D, E, G, A, B	LM	DS	(Haussmann et al. 2002)
Total leaf area at seedling	141 RILs (B923296 and SC170-6–8) and 44 diverse inbred lines	337	DArT	8	LM	–	(Mace et al. 2012)
Green leaf area at maturity	245 RILs (M35-1 and B35)	237		2, 3, 9	LM	DS	(Reddy et al. 2014)
	168 RILs (296B and IS18551)	152	SSR,	1, 6	LM	DS	(Srinivas et al. 2009)
	648 SC lines and Chromatin breeding lines and hybrids	131,544	SNP, morphological		GWAS	DS	(Spindel et al. 2018)
Flag leaf area	70 RILs (Tx430 and Tx7078)	261	SNP	6, 1	LM	DS	(Kapanigowda et al. 2014)
	70 RILs (Tx430 and Tx7078)	261	SNP	7	LM	DS	(Kapanigowda et al. 2014)
Percent green leaf area at maturity	245 RILs (M35-1 and B35)	237		2, 3	LM	DS	(Reddy et al. 2014)
	168 RILs (296B and IS18551)	152	SSR, morphological	3, 9	LM	DS	(Srinivas et al. 2009)
Rate of leaf senescence	245 RILs (M35-1 and B35)	237	SSR, morphological	10	LM	DS	(Reddy et al. 2014)
Grain yield per panicle	245 RILs (M35-1 and B35)	237	SSR, morphological	3, 4, 6, 9	LM	DS	(Reddy et al. 2014)
	200 MAGIC	79,728	SNP	1, 5, 7	GWAS	DS	(Ongom 2016)
	248 RILs (Tx436 and 00MN7645)	7144	SNP	1, 6, 8	GWAS	DS	(Sukumaran et al. 2016)
	184 F8 RILs (E36-1 × SPV70)	104	EST-SSR, SSR, SNP	3, 8	LM	DS	(Fakrudin et al. 2013)
	100 (BR007 and SC283)	344	DArT, SSR, STS	2, 3, 6, 8, 10	LM	DS	(Sabadin et al. 2012)
Stress tolerance index	200 MAGIC	79,728	SNP	6, 1, 8, 9	GWAS	DS	(Ongom 2016)
CO2 assimilation rate (A)	70 RILs (Tx430 and Tx7078 F6)	261	SNP	1, 5, 9	LM	DS	(Kapanigowda et al. 2014)
Transpiration rate (E)	70 RILs (Tx430 and Tx7078 F6)	261	SNP	1, 7	LM	DS	(Kapanigowda et al. 2014)
A:E ratio	70 RILs (Tx430 and Tx7078 F6)	261	SNP	6, 9, 10	LM	DS	(Kapanigowda et al. 2014)
Stomatal conductance	28,107 (Early HegariSart and BK7)	6128	SNP	7, 10	LM	DS	(Lopez et al. 2017)
Stomatal density	70 RILs (Tx430 and Tx7078)	6128	SNP	2, 7	LM	DS	(Lopez et al. 2017)
	70 RILs (Tx430 and Tx7078 F6)	261	SNP	7	LM	DS	(Kapanigowda et al. 2014)
Nodal root angle	141 RILs (B923296 and SC170-6-8) and 44 diverse inbred lines	337	DArT	5, 8, 10	LM	–	(Mace et al. 2012)
Root dry weight	141 RILs (B923296 and SC170-6-8) and 44 diverse inbred lines	337	DArT	2, 5, 8	LM	–	(Mace et al. 2012)
	184 F8 RILs (E36-1 × SPV70)	104	EST-SSR, SSR, SNP	4	LM	DS	(Fakrudin et al. 2013)
Leaf drying after drought	107 (Sorghum association panel)	98	SSR	1, 3	GWAS	DS	(Sakhi et al. 2013)
leaf and stem biomass	70 RILs (Tx430 and Tx7078 F6)	261	SNP	6	LM	DS	(Kapanigowda et al. 2014)
Root fresh weight	184 RILs (E36-1 × SPV70)	104	EST-SSR, SSR, SNP	4	LM	DS	(Fakrudin et al. 2013)
Shoot dry weight	141 RILs (B923296 and SC170-6-8) and 44 diverse inbred lines	337	DArT	1, 5	LM	–	(Mace et al. 2012)
Root to shoot ratio	141 RILs (B923296 and SC170-6-8) and 44 diverse inbred lines	104	EST-SSR, SSR, SNP	10	LM	DS	(Fakrudin et al. 2013)
Root length (cm)	184 F8 RILs (E36-1 × SPV70)	104	EST-SSR, SSR, SNP	4	LM	DS	(Fakrudin et al. 2013)
Root volume	184 RILs (E36-1 × SPV70)	104	EST-SSR, SSR, SNP	1, 4	LM	DS	(Fakrudin et al. 2013)
Number of roots/plant	184 RILs (E36-1 × SPV70)	104	EST-SSR, SSR, SNP	10	LM	DS	(Fakrudin et al. 2013)
Crown root angle, mature	28,107 RILs (Early HegariSart and BK7)	6128	SNP	3	LM	DS	(Lopez et al. 2017)
Nodes with brace roots	611 RILs (Sansui and Jiliang)	109	SSR	6, 7	LM	DS	(Li et al. 2014)

QL39 = Drought susceptible but midge-resistant line; QL41 = Stay green drought tolerant derived from the cross QL33/B35; Tx436 = Food grain type; 00MN7645 = drought tolerant; M35-1 = Drought susceptible; B923296 = narrow nodal root angle nodal; SC170-6–8 = wide nodal root angle; E36-1 = high yielding line from guinea-caudatum hybrid race with Ethiopian origin, well adapted to tropical environment and has thin and short roots; SPV570 = Good grain and fodder quality, a promising restorer line on Milo cytoplasm and has the thick and long roots. TX7078 = Pre-flowering-tolerant, post-flowering susceptible; B35 = Pre-flowering susceptible, post-flowering-tolerant, Tx430 = high yielding, susceptible to post flowering drought stress; RT37000 = Senescent; T7000 = pre-flowering-tolerant, post-flowering susceptible and high yielding, sensitive to lodging; SC56 = Caudatum-nigricans from Sudan, is a post-flowering drought-tolerant (stay green) and lodging-tolerant line, but susceptible to pre-flowering drought stress. E36-1, the source for the stay-green trait is a high-yielding breeding line assigned to the guinea-caudatum hybrid race with Ethiopian origin. Line IS9830 is a tall Sudanese feterita belonging to the caudatum race. Line N13 from India is a durra sorghum

^a^LM = Linkage mapping, GWAS = Genome-wide association study

^b^Env = Environment, DS = Drought stress, RF = Rainfed

### Mapping stay green and chlorophyll content

Various drought-tolerant sorghum genotypes have been identified, which include *00MN7645* (Sukumaran et al. 2016), *QL41* (Tao et al. 2000), *B35* (Crasta et al. 1999; Subudhi et al. 2000; Xu et al. 2000), *BTx642* (Harris et al. 2007), *SC-56* (Kebede et al. 2001), and *E-36-1* (Haussmann et al. 2002). These genotypes have served as sources of drought tolerance genes, and used as parents to develop recombinant inbreed lines (RILs) and near-isogenic line (NILs) mapping populations for identification of QTLs encompassing the stay green genes. Through this approach, several QTLs associated with stay green have been identified in sorghum using different markers (Sabadin et al. 2012; Sukumaran et al. 2016) (Table 2). Among the known drought-tolerant sorghum genotypes, *B35,* which is derived from genotype *BTx642* (a durra sorghum from Ethiopia), has been used as a major source of stay green genes in sorghum breeding programs aimed at improving its drought tolerance, in the United States, Australia, India and other parts of the world (Evans et al. 2013).

Using 98 RILs developed from a cross between *TX7078* (pre-flowering-tolerant, post-flowering susceptible) and *B35* (pre-flowering susceptible, post-flowering-tolerant), (Tuinstra et al. 1997) identified three QTLs associated with stay green. Three major stay-green QTLs that explained 42% of the total phenotypic variance were identified using 96 RILs derived from a B35 × Tx430 cross (Crasta et al. 1999). In another study, four QTLs (*Stg1*, *Stg2*, *Stg3*, and *Stg4*) associated with stay green were identified using 98 RILs (derived from B35 × Tx7000 cross) grown under post-flowering drought stress conditions (Xu et al. 2000). Subsequently, by planting the same genotypes for 2 years at two sites, Subudhi et al. (2000) confirmed that four of these stay green QTLs (*Stg1*, *Stg2*, *Stg3* and *Stg4*) are indeed associated with the trait. Similarly, using NILs mapping population derived from BTx642 × RTx7000, alleles that contribute to stay green were mapped to the four major QTLs (*Stg1*, *Stg2*, *Stg3* and *Stg4*) (Harris et al. 2007). These QTLs have been introduced in several genetic backgrounds through marker-assisted breeding (MAB) and were shown to enhance post-flowering drought tolerance (Kassahun et al. 2010; Kamal et al. 2017).

The comparison of the four stay green QTL profiles showed that *Stg1*, *Stg2* and *Stg3* are more important contributors to the expression of stay green trait, as they explained higher phenotypic variance and showed consistency across different genetic backgrounds (Subudhi et al. 2000). *Stg1* and *Stg2*, which accounted for 20% and 30% of the phenotypic variance, respectively, were mapped to SBI-03 while *Stg3* and *Stg4,* which explained 16% and 10% of the phenotypic variance, respectively, were mapped to SBI-02 and SBI-05 in that order (Xu et al. 2000; Sanchez et al. 2002). Furthermore, the *Stg1* and *Stg2* regions also contain genes responsible for key enzyme regulating photosynthesis, heat shock proteins (HSPs) and abscisic acid (ABA), which are important for drought and heat stress tolerance and grain yield in sorghum (Xu et al. 2000). Further characterization of these stay green QTL regions may lead to discovering new genes that will deepen our understanding about drought tolerance mechanisms, which in turn facilitates the manipulation of the stay green trait in sorghum and other cereal crops.

The chlorophyll content SPAD readings at booting (SPADB) and maturity (SPADM) were associated with stay green ratings in sorghum (Xu et al. 2000). QTLs for chlorophyll content SPAD readings overlapped with QTLs for stay green under drought stress conditions (Xu et al. 2000; Harris et al. 2007; Borrell et al. 2014b). This indicates the possibility of developing drought-tolerant sorghum cultivars combining these important traits. Three QTLs (*Chl1*, *Chl2* and *Chl3*) explaining 20–30% of the phenotypic variability in chlorophyll content were identified through exposing 98 sorghum RILs to post-flowering drought stress (Xu et al. 2000). All the three QTLs overlapped with the three stay green QTL regions (*Stg1*, *Stg2* and *Stg3*) and accounted for 46% of phenotypic variance (Xu et al. 2000) suggesting that these traits may be at least partly regulated by the same genes.

Several QTLs linked to chlorophyll SPAD were identified in different studies. For instance, Reddy et al. (2014) identified 7 QTLs in 245 RILs, including major QTLs on SBI-09 and SBI-10 accounting for 15% of the total SPADB variation and 14.1% of SPADM variation, respectively. QTLs linked to chlorophyll contents measured at three different stages of plant growth were also found (Sukumaran et al. 2016; Gelli et al. 2017). In another study, a QTL associated with chlorophyll concentration in the flag leaf, which explained 13% of the phenotypic variance was detected on SBI-04 (Kapanigowda et al. 2014). The results suggest that through transferring favorable alleles representing these QTLs, improving chlorophyll content of drought sensitive sorghum genotypes is possible using crossbreeding and marker assisted selection approaches.

### Genes involved in drought stress tolerance

The transcriptional response of sorghum exposed to drought stress includes sets of differentially expressed gene products, such as Late Embryogenesis Abundant (LEA) proteins, Delta 1-pyrroline-5-carboxylate synthase (P5CS2), high-affinity K^+^ transporter 1 (HKT1) and proteins associated with response to ABAs (Johnson et al. 2014). Genes encoding a dehydration-responsive element-binding (DREB1A) transcription factor, salt and drought-induced RING finger 1 (SDIR1) and a CBL interacting serine/threonine-protein kinase 1 (CIPK1), trehalose-6-phosphate synthase (TPS) and P5CS2 was highly expressed in the stay-green line compared to the senescent line (Johnson et al. 2015). The increased expression of *P5CS2* gene in the stay-green line was associated with higher proline levels and is localized in *Stg1* QTL region Johnson et al. (2015), which was previously identified as stay-green QTL (Subudhi et al. 2000; Xu et al. 2000). Furthermore, a comparative transcriptome study on sorghum genotypes with contrasting WUE in response to drought revealed higher number of differentially expressed genes (in “response to stress” and “abiotic stimulus”, as well as for “oxidation–reduction reaction) in the sensitive genotype (Fracasso et al. 2016). The transcriptome analysis on sorghum seedlings exposed to drought stress for 1 h (early) and 6 h (late) identified early and the late responsive genes for drought tolerance, as well as genes expressed only in drought-tolerant genotypes (Abdel-Ghany et al. 2020). Two ethylene-responsive transcription factor (ERF) genes were upregulated under mild and severe drought stress conditions and down-regulated under re-watering treatment (Zhang et al. 2019a).

Transcripts encoding for the mitochondrial Transcription tERmination Factor (mTERF) family, anion-transporting ATPase family proteins and LEA, hydroxyproline-rich glycoprotein family protein were highly up-regulated under mild stress whereas proteins with putative homology to ABscisic acid- Insensitive 2 (ABI2) and mannosyltransferase were among the most highly elevated during severe drought stress (Devnarain et al. 2019). Moreover, major transcription factors including heat stress transcription factor (HSF), ethylene-responsive transcription factor (ERF), Petunia NAM, Arabidopsis ATAF1/2 and CUC2 (NAC), WRKY transcription factor (WRKY), homeodomain leucine zipper transcription factor (HD-ZIP) were highly upregulated under drought stress conditions (Varoquaux et al. 2019). Genes encoding for heat shock protein (HSPs), LEAs, chaperones, aquaporins, and expansins might play important roles in sorghum drought tolerance (Varoquaux et al. 2019). Upregulation of the above-mentioned genes under drought stress indicates their potential role in drought tolerance. These genes could be important targets for improvement of drought tolerance in sorghum and other cereals.

APETALA2-Ethylene Responsive Factors (AP2-ERFs) are plant-specific transcriptional regulators characterized by one or more DNA binding AP2/ERF domains. AP2/ERFBP TFs perform diverse roles in plant biological processes, such as cell proliferation, vegetative and reproductive development, plant hormone and abiotic/biotic stress responses (Sharoni et al. 2011; Xu et al. 2011). A genome-wide analysis of the ERF gene family in sorghum, identified 105 sorghum ERF (SbERF) genes (Nakano et al. 2006). In another study, 158 ERF genes with 52 of them encoding DREB while 106 code for ERF subfamily proteins were reported in sorghum (Mathur et al. 2020). Genes encoding DREBs, AP2/ERF and MYB transcription factors (TFs) are amongst the early response genes in sorghum when plants are stressed under PEG (Abdel-Ghany et al. 2020). The DREB1A transcription factor gene was also expressed at higher levels in a stay green sorghum line (Johnson et al. 2015). Interestingly, sorghum DREB2 expression in rice improved both tolerance and yield under drought stress (Bihani et al. 2011). In response to severe stress, WRKY transcription factor gene was highly over-expressed in sorghum (Devnarain et al. 2016) and these transcription factors were largely inhibited at pre-flowering stress, but less so to post-flowering drought stress in sorghum root samples (Varoquaux et al. 2019).

## Proteome response to drought stress in sorghum

Genes are better characterized by proteins they encode that directly recognize relevant stresses, activate signaling transduction, and regulate the expression and translocation of the proteins required for responding to the stresses. Proteomics studies are therefore crucial to understand plant response and identify key proteins that determine the outcomes of plant response to particular stresses. As such, proteomics studies have received increasing attention to deepen our understanding at molecular-level with regard to sorghum response to drought stress (Ngara et al. 2021). The proteomic analysis of drought tolerant (El9) and sensitive sorghum genotypes exposed to drought stress identified proteins only expressed in drought-tolerant genotype, such as HSPs, GrpE protein homolog and Glycine-rich RNA-binding protein (GR-RBP) (Fadoul et al. 2018). HSP is regulated by stress inducible protein called GrpE protein, and its expression under drought stress conditions were reported in different crops (Sato and Yokoya 2008; Kim et al. 2015; Piveta et al. 2021). Under drought stress, GR-RBP was reported to involve in the regulation of ABA and stress responses and also play a role in RNA transcription (Kim et al. 2010). In another study, multiple HPSs and dehydrins were significantly upregulated in pre-flowering drought tolerant (RTx430) and sensitive (BTx642) sorghum genotypes grown under drought stressed field conditions (Ogden et al. 2020). The chloroplastic form of HSP 60 and disulfide isomerase were also upregulated in drought-tolerant genotype after 24 h of recovery following exposure to drought stress (Jedmowski et al. 2014).

The protein profile of organelles and cytosolic response for drought stress were different and the drought response of organelles may be more genotype-specific compared to that of cytosol. A study by Ogden et al. (2020) showed that organelle-localized proteins such as proteins associated with ABA metabolism and signal transduction, Rubisco activation, reactive oxygen species scavenging, flowering time regulation, and epicuticular wax production were only upregulated in the drought-tolerant genotypes. The protein profiles of cytosolic and organelle-enriched cellular compartments in sorghum genotypes with regard to the plants’ response to drought stress have not been well studied. Such studies can definitely generate interesting information on the mechanism of drought tolerance in sorghum and other crops. ABA is a well-characterized stress hormone typically synthesized de novo in response to drought (Roychoudhury et al. 2013; Sah et al. 2016).

Root proteomic profiles of two sorghum genotypes responding to water stress showed that both genotypes raised the expression level of thioredoxins, peroxidases, glutathione-s-transferase and germins, which are vital in reactive oxygen species (ROS) metabolism. Eleven transcription-related proteins (six histone proteins and three nascent polypeptide-associated complex (NAC) subunit beta proteins were expressed in root proteome of only drought-tolerant genotype in response to water deficit condition (Goche et al. 2020). NAC is one of the most important TF families involved in plant senescence and response to drought stress (Guo et al. 2004; Tran et al. 2004; Wu et al. 2016) and other biotic and abiotic stresses (Fujita et al. 2004). Expression analysis of NAC transcription factors gene family in sorghum identified 13 proteins involved in drought stress tolerance at the post-flowering stage in sorghum (Sanjari et al. 2019) suggesting their involvement in plant response to drought stress. Hence, studies in sorghum that involve their overexpression and functional characterization could shed more light on the role of NAC genes in plant response to drought stress. The proteomic analyses of drought stress response in sorghum root at the seedling stage, revealed that proteins associated with changes in energy usage; osmotic adjustment; ROS scavenging; and protein synthesis, processing, and proteolysis play important roles in maintaining root growth under drought stress (Li et al. 2020). Ribulose Bisphosphate Carboxylase (RuBisCo), Oxygen-evolving enhancer protein, Acidic endochitinase, a defense- and immunity-related proteins were upregulated in the sorghum Btx642 leaf tissue under post-flowering drought stress (Woldesemayat et al. 2018) suggesting their involvement in the plant response to drought stress.

Rubisco is a vital photosynthetic enzyme responsible for CO_2_ fixation (Fernie and Bauwe 2020). If the rubisco catalytic activity is ineffective, the photosynthesis capacity of a plant become limited (Galmés et al. 2019). The transfer of sorghum small subunit Rubisco (RbcS) considerably boosted catalytic rate of Rubisco in transgenic rice (Ishikawa et al. 2011). The higher photosynthetic rate under elevated CO_2_ conditions in rice were observed in the overexpression of RbcS sorghum and knocked-out rice RbcS by CRISPR/Cas9 system (Matsumura et al. 2020). Sorghum Rubisco has a considerably higher catalytic rate and comparatively high amino acid sequence identity to that of rice (Fukayama et al. 2019). Given the results of these studies, sorghum may serve as a novel source of proteins that boost photosynthetic efficiency and increase crop yield under forthcoming elevated CO_2_ levels due to its high activity-type Rubisco. Sufficient information is not yet available on sorghum genetic variation in terms of catalytic rate of rubisco. Hence, identification of sorghum genotypes that differ in their rubisco catalytic rates followed by their genetic analyses is important for deeper understanding of sorghum rubisco in relation to that of other crops. In addition, overexpression of sorghum rubisco genes in other crops could prove helpful in coping with drought stress and improving productivity.

## Combined effects of drought stress and other major biotic and abiotic factors on plant growth and development

### Drought interaction with other abiotic stresses

Multitude of stress factors, which may aggravate the effects of drought-induced stress or enhance plant tolerance, continuously challenge plants growing under natural conditions. Several abiotic stresses such as nutrient deficiency, aluminum toxicity, water logging, salinity, and low and high temperature stresses (Tari et al. 2013) are known to negatively affect sorghum grain yield and quality. Understanding the effects of these stresses occurring concomitantly with drought stress is crucial to optimize management strategies, induce drought stress tolerance, and accelerate sorghum breeding for enhancing tolerance to these stresses.

It has been shown that elevated CO_2_ concentration reduced stomatal conductance that allows maintained whole-plant metabolism and enhanced grain protein content in drought stressed sorghum (De Souza et al. 2015). This indicates that sorghum is a resilient crop that can maintain its importance as a food security crop for subsistence farmers in the face of climate change. However, only two levels of CO_2_ concentrations were used and the study was not conducted across contrasting environments to address the variation due to other environmental factors that might have affected the interactions between drought and heat stresses. Evaluation of drought and heat stresses imposed at different growth stages of sorghum affects ethanol production (Ananda et al. 2011). Similarly, Impa et al. (2019) studied the effects of drought and heat stresses separately and showed that both affect sorghum yield and nutritional quality. Although drought and heat are prominent stresses in sorghum production, in the studies by Impa et al. (2019) and Ananda et al. (2011), the sorghum genotypes were grown at two environments that received either drought or heat stress, and thus it is not possible to make inferences about the interactions between the two stresses.

Simultaneous application of drought and heat stresses reduced soil water content (SWC), leaf relative water content (RWC), leaf water potential (Ψ), and leaf osmotic potential (π) in sorghum (Machado and Paulsen 2001). The co-occurrence of these stresses may have a greater negative impact on the plants as compared to their separate effects. Both drought and heat stresses affect functional biochemistry and reduce grain yield and nutritional quality of several crops including sorghum (Sehgal et al. 2018). A study by Hattori et al. (2005) showed that application of silicon enhanced root growth, maintained photosynthetic rate and stomatal conductance in sorghum cultivars grown under drought stress conditions, with varying level of drought tolerance. This indicates that soil silicon amendments can minimize the effects of drought on plant growth and development. Drought stress during post-flowering stage increased susceptibility to charcoal rot and water lodging, as reviewed in Burke et al. (2018). The severity of stalk and charcoal rot disease in sorghum plants was lower under drought stress conditions when compared with the case in well-irrigated plants (Kapanigowda et al. 2013). These results suggest that interaction between drought and other abiotic stresses is complex, particularly under natural conditions as it involves several factors. However, the presence of sorghum genotypes that are tolerant to drought and other abiotic stresses (Burke et al. 2018) indicates the possibility of developing cultivars combining these important traits.

### Microbes-induced drought stress tolerance in sorghum

Domestication of crops has led to the loss of genetic diversity in plants and the microorganisms associated with them (Perez-Jaramillo et al. 2016). Many studies have demonstrated that microorganisms found in nature can improve plant health, disease resistance, tolerance to abiotic stresses and increase yields (Trivedi et al. 2020). Moreover, it is also shown that the plant genotypes determine microbiomes to be recruited (Wagner et al. 2016). The interplay of a specific plant genotype with its microbiome is fundamental for their fitness by buffering environmental constraints. However, current sorghum genotypes and varieties may not have been developed for utilizing the beneficial impact of microorganisms found in nature. There is thus a tremendous potential of microbes to explore for inducing drought tolerance for sorghum cultivation. Studies by Carlson et al. (2020) have demonstrated that the addition of rhizobacteria to sorghum seedlings induced systemic tolerance to drought by the early activation of signaling hormones such as brassinolides, salicylic acid and jasmonic acid. This study also hinted about the possibility of bacterial ACC deaminase lowering plant ethylene levels through cleaving ACC into α-ketobutyrate and ammonia and promoting plant growth under adverse conditions. Therefore, modulation of the sorghum microbiome can be one strategy for overcoming drought stress. As a first step before microbiome manipulation, it is essential to understand the sorghum-associated microbiomes under different stresses. Xu et al. (2018) observed the increased presence of monoderm bacteria with a thick cell wall that lacks an outer cell membrane and positively influences plant growth in drought-stressed sorghum. The possible explanation for the increased abundance of monoderm bacteria microbiome in drought-stressed sorghum was the exudation of a specific plant metabolite, glycerol-3-phosphate (G3P), an essential precursor to peptidoglycan biosynthesis of monoderm bacteria. Furthermore, monoderm bacteria such as Actinobacteria were enriched in drought and heat stresses, affecting plant development positively, further stressing the importance of the microbiome in drought tolerance (Wipf et al. 2021). Furthermore, studies are needed to understand the role of fungal communities in drought tolerance in sorghum and their interplay with bacterial communities. To harness the sorghum microbiomes for drought-resilient sorghum production, it is vital to focus on longer-term experiments under field conditions for an improved mechanistic understanding of the complex relationships between sorghum and microbes during drought conditions.

## Conclusion and future work

Drought is a key limiting factor in major sorghum growing areas, which substantially reduces the productivity and hence production of the crop. In the face of climate change and decreasing water availability, the effects of drought stress imposed at different growth and developmental stages on grain yield and quality of sorghum are well established. Although drought stress spanning across several plant developmental stages is a common occurrence in major sorghum growing areas, most studies have focused only on the effect of drought stress that occur during specific plant growth stages. Hence, in order to gain a more definitive understanding of the overall effect of drought on sorghum and the characteristics of plant responses to drought, it is imperative to conduct well-planned and detailed studies that cover the entire plant growth and development stages. Overall, developing drought-tolerant cultivars that suit a range of agro-climatic conditions, especially in the arid and semi-arid regions, is crucial to avert the negative impacts of drought stress. In this regard, the identification of sorghum genotypes with strong tolerance to drought stress is the first step towards the development of drought-tolerant cultivars bearing various desirable characteristics. However, identification of acceptable drought-tolerant genotypes requires well-planned studies that represent spatiotemporal patterns, variable intensity and duration of drought stress during crop growing season, and variation in edaphic conditions across major sorghum growing areas. Dissecting molecular mechanisms of drought tolerance using genetics, genomics, proteomics and metabolomics studies would lead to the identification of molecular signatures that can be targeted for improving the drought tolerance of desirable sorghum germplasm using molecular breeding techniques.

### *Author contribution statement*

KBA, ME, AC and MG contributed to conceptualization of the manuscript. KBA, ME and RV wrote the draft manuscript. DN and TM provided the sorghum nutritional content data. MG, AC, TF, DN and TM edited the manuscript and contributed to the revision process. All authors read and approved the submitted version.

## Data Availability

The datasets analyzed during the current study are available from the corresponding author on reasonable request.
